# Enhanced functional connectivity between habenula and salience network in medication-overuse headache complicating chronic migraine positions it within the addiction disorders: an ICA-based resting-state fMRI study

**DOI:** 10.1186/s10194-021-01318-3

**Published:** 2021-09-09

**Authors:** Wei Dai, Enchao Qiu, Yun Chen, Xinbo Xing, Wei Xi, Meichen Zhang, Ke Li, Lixia Tian, Zhao Dong, Shengyuan Yu

**Affiliations:** 1 Department of Neurology, Chinese PLA General Hospital, 28 Fuxing Road, 100853 Beijing, China; 2 Chinese PLA Medical School, 100853 Beijing, China; 3 Department of Radiology, Fourth Medical Center of Chinese PLA General Hospital, 100048 Beijing, China; 4 School of Computer and Information Technology, Beijing Jiaotong University, 100044 Beijing, China

**Keywords:** Medication-overuse headache, Migraine, Salience network, Habenula, Resting-state fMRI

## Abstract

**Background:**

Medication-overuse headache (MOH) is a relatively frequently occurring secondary headache caused by overuse of analgesics and/or acute migraine medications. It is believed that MOH is associated with dependence behaviors and substance addiction, in which the salience network (SN) and the habenula may play an important role. This study aims to investigate the resting-state (RS) functional connectivity between the habenula and the SN in patients with MOH complicating chronic migraine (CM) compared with those with episodic migraine (EM) and healthy controls (HC).

**Methods:**

RS-fMRI and 3-dimensional T1-weighted images of 17 patients with MOH + CM, 18 patients with EM and 30 matched healthy HC were obtained. The RS-fMRI data were analyzed using the independent component analysis (ICA) method to investigate the group differences of functional connectivity between the habenula and the SN in three groups. Correlation analysis was performed thereafter with all clinical variables by Pearson correlation.

**Results:**

Increased functional connectivity between bilateral habenula and SN was detected in patients with MOH + CM compared with patients with EM and HC respectively. Correlation analysis showed significant correlation between medication overuse duration and habenula-SN connectivity in MOH + CM patients.

**Conclusions:**

The current study supported MOH to be lying within a spectrum of dependence and addiction disorder. The enhanced functional connectivity of the habenula with SN may correlate to the development or chronification of MOH. Furthermore, the habenula may be an indicator or treatment target for MOH for its integrative role involved in multiple aspects of MOH.

## Background

Medication-overuse headache (MOH) is a secondary chronic disorder attributed to overuse of acute or symptomatic headache medications that develops in patients with a pre-existing primary headache, especially migraine [1, 2]. The 1-year relapse rate of MOH patients could reach to 24.8 %, and two-thirds of MOH patients fulfill the criteria for substance addiction [3–5]. Neuroimaging, genetic and biological studies indicated that MOH shares similar imaging features, associated gene polymorphisms, and neurobiological changes with substance addiction [6–10]. In addition, MOH patients can develop behaviors including ritualized drugs intake, psychological drug attachment and withdraw symptoms which are believed to be associated with addiction [11–13].

Previous resting-state functional magnetic resonance imaging (RS-fMRI) method has indicated dysregulation of the reward circuit in MOH patients in line with substance abusers [14, 15]. And alterations of the functional connectivity between the nucleus accumbens and the salience network (SN) as well as other brain regions in the reward-related circuits have been reported [16]. Besides the reward system, addiction is also associated with the enhanced activation of the anti-reward circuit, in which the habenula plays a key role [17]. The habenula functions in addiction, cognition, reward, pain and analgesia while the SN is to modulate information via integrating sensory, affective and cognitive inputs, and assigning relevance of the stimuli for continuous processing [18–21]. However, few studies to date have focused on habenula and its interaction with the SN.

**Fig. 1:**
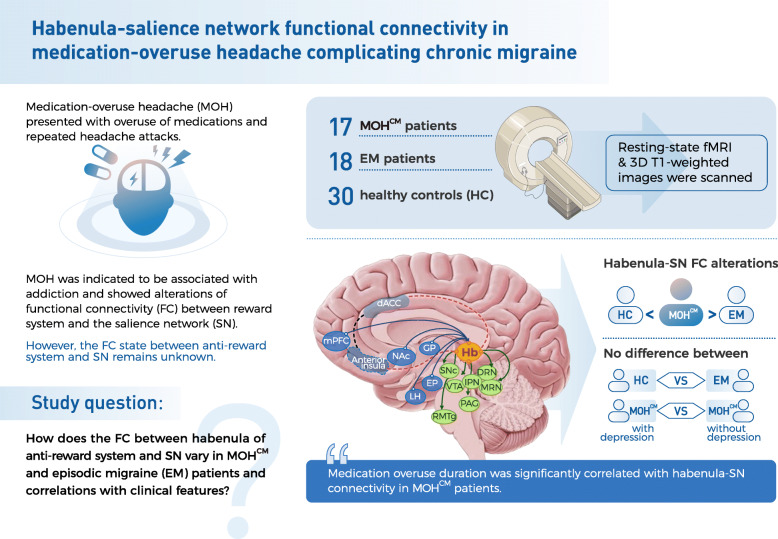
Summary of the current study. This figure is a concise summary of the current study. MOH + CM (medication-overuse headache + chronic migraine) was shown as MOH^CM^ here for a better layout in this figure. The picture of brain on the right shows the habenula and its connections with other brain regions. The full lines with arrows connect regions which the habenula has afferent inputs from or efferent outputs to on structural basis. The dotted lines represent functional connectivity identified by fMRI studies. Hb, habenula; SNc, substantia nigra pars; IPN, interpeduncular nucleus; DRN, dorsal raphe nucleus; MRN, median raphe nucleus; VTA, ventral tegmental area; PAG, periaqueductal gray; RMTg, rostromedial tegmental nucleus; EP, entopeduncular nucleus; NAc, nucleus accumbens; CP, caudate/putamen; LH, lateral hypothalamic; dACC, dorsal anterior cingulate cortex; mPFC, medial prefrontal cortex

Therefore, this study aims to investigate the resting-state function connectivity of the habenula and the SN in patients with MOH complicating chronic migraine (CM) compared with those with episodic migraine (EM) and healthy controls (HC). And we also searched for possible correlations between these functional connectivity alterations and clinical features (Fig. 1).

## Materials and methods

### Participants

All research procedures were approved by the Chinese Ministry of Health and the Ethics Committee of the Chinese PLA General Hospital, Beijing, China, which were conducted in accordance with the ethical principles of the Declaration of Helsinki. Participants were recruited in Chinese PLA General Hospital and received complete description of the study and gave written informed consent before the study.

Patients were recruited consecutively in the International Headache Center in the Chinese PLA General Hospital from May 2018 to April 2019. All HC had no primary headaches or other chronic pain disorders. The included criteria of MOH and EM were as follows: (1) The diagnosis of MOH and EM met the criteria of the International Classification of Headache Disorders 3rd edition (ICHD-3) [22]; (2) All MOH patients were confirmed to be with a prior migraine based on their past history. Thus, according to ICHD-3, these patients should be given the diagnosis of MOH + chronic migraine (CM); (3) EM patients were excluded if they had suffered a prior episode of MOH; (4) Without migraine preventive medication in the past 3 months. The excluded criteria were as follows: (1) With chronic disorders including hypertension, diabetes mellitus, cardiovascular diseases, ischemia etc.; (2) With cranium trauma, psychotic disorder, or regular use of a psychoactive or hormone medication. All participants were right hand dominant and reported no other neurologic or psychiatric disorder. The evaluations of participants were conducted by 2 neurologists. Meanwhile clinical data as well as the Patient Health Questionnaire 9 Depression Scale (PHQ-9) and Generalized Anxiety Disorder Assessment 7-item Scale (GAD-7) were collected before MRI scan. MRI scans were taken at least 24 h after the latest migraine attack for MOH or EM patients. The alcohol, nicotine, caffeine and other substances were avoided at least 24 h before MRI examination.

### MRI data acquisition

All MRI studies were performed on a 3.0T Siemens scanner. The RS-fMRI were acquired right after localizer images. Then whole-brain 3-dimensional T1-weighted images of each one was then obtained as sagittal view (repetition time = 2500 ms, echo time = 3.5 ms, flip angle = 8°, field of view = 256 × 256 mm^2^, Matrix = 256 × 256, slices = 192, thickness = 1 mm, interslice gap = 0.6 mm). RS-fMRI were obtained using a gradient echo-planar imaging (EPI) sequence (repetition time = 2000 ms, echo time = 30 ms, flip angle = 90^◦^, slice thickness = 3 mm, slice gap = 0.6 mm, field of view = 200 × 200 mm^2^, Matrix = 64 × 64). For all of the participants, the structural images were examined to exclude the possibility of lesions by 2 radiologists.

### RS-fMRI data preprocessing

The RS-fMRI data were preprocessing using the FMRIB’s Software Library (FSL) tools (http://www.fmrib.ox.ac.uk/fsl/)[23].The RS-fMRI data were processed by the following steps as performed in previous studies [24–26]. They were: (1) removing the first 5 volumes, (2) correcting the head motion by MCFLIRT, (3) removing the nonbrain tissues by BET (Brain Extraction Tool), (4) spatial smoothing using a 4-mm FWHM (full width at half maximum) Gaussian kernel, (5) removing slow drift with a high-pass temporal filter of 0.01 Hz, and (6) registering the RS-fMRI data to the 3-dimensional T1-weighted images and then to Montreal Neurological Institute-152 standard space. The registered RS-fMRI data were finally resampled to 2 × 2 × 2 mm.

### TC-GICA and dual-regression

The temporal-concatenation group independent component analysis (TC-GICA) on the datasets of 30 healthy volunteers were created using the MELODIC (Multivariate Exploratory Linear Optimized Decomposition into Independent Components) tool in FSL [27]. The number of components was fixed at 50. Then we built individual-subject-level spatial maps by applying dual regression to the preprocessed RS-fMRI data of each participant by using the same approach. For each subject, firstly we used the previously obtained GICA spatial maps to regress against the individual’s preprocessed RS-fMRI data to estimate the matrices of network time series of each component. Secondly, the 50 network time series which we obtained were regressed against each subject’s preprocessed 4-dimensional RS-fMRI data to estimate the subject’s network spatial component maps. The independents were demeaned and normalized in both of the 2 steps before being entered into the regression model [25, 28]. Then we used FSL utility to spatially correlate all the components to Yeo’s 7 network parcellation of cortex with the results of r-value [29]. Each component of a resting-state network was ensured that the bulk of its signal falls on the lower end of the power frequency spectrum. Visual inspections were performed as a final check.

### Habenula and SN interaction difference analyses

In our study, we tested the alterations of habenula and SN interaction in patients with MOH + CM and EM. Based on the spatial maps generated by TC-GICA, we identified SN as follows, mainly encompasses the dorsal anterior cingulate cortex (dACC) and bilateral anterior insula/frontal operculum [21].Then we got the SN spatial maps which corresponded to each brain network across individuals in a 4-dimensional file. And we compared three group of MOH + CM vs. EM vs. HC using ANOVA in DPABI toolkit under MATLABR2013b (version 8.2.0.701) environment, and subsequent LSD test to compare the difference between each two groups [30]. PHQ-9 and GAD-7 scale were added as covariates when ANOVA were performed to exclude the effects of depression or anxiety. Finally, we performed FDR (false discovery rate) procedure to correct for multiple comparisons within habenula mask. The habenula masks were manually created for every patient, by using the T1 images to visually identify the right and left habenula of each subject landmark using SPM8 (www.fil.ion.ucl.ac.uk/spm/). In T1 images the habenula is visible as two small epithalamic structures at the dorsomedial portion of thalamus pointing into the third ventricle [31]. All the habenula masks were carefully inspected, and the mean MNI coordinates of all the participant-specific habenula masks were approximately centered at [− 4, − 24, 2] for the left habenula and [4, − 24, 2] for the right habenula which were consistent with previous research [31]. The habenula masks were defined as a 4-mm sphere centered at [− 4, − 24, 2] and [4, − 24, 2]. As for MOH + CM and EM, each group will be divided into two subgroups based on PHQ-9 scale (subgroup 1 with scores 0–4 and subgroup 2 with scores 5–27), or GAD-7 scale (subgroup 1 with scores 0–4 and subgroup 2 with scores 5–27) to detect if there is a significant difference between the subgroups in MOH + CM or EM. Comparisons of functional connectivity will be performed between two subgroups in order to validate if depression or anxiety affects the functional connectivity.

### Statistical analysis

Analyses of variance with subsequent post-hoc tests, Chi-square tests, or Fisher’s exact tests were used as appropriate for comparing demographic data among participant cohorts. Pearson correlation analysis was performed between functional connectivity of habenula-SN in the imaging and the clinical variables within the patients’ group using SPSS. The data with normal distribution was described as mean ± standard deviation. *P* value of < 0.05 was considered to indicate a statistically significant correlation.

## Results

### Participant characteristics

Table 1 shows the demographic and clinical characteristics of the subjects included in this study. Sixty-five subjects were included in the RS-fMRI analysis. Seventeen patients with MOH + CM (3 males and 14 females, mean age 46.6 ± 10.3 years), 18 patients with EM (4 males and 14 females, mean age 38.0 ± 14.4 years) and 30 HC (11 males and 19 females, mean age 40.8 ± 9.7 years). Age and sex did not differ across groups. Disease duration of MOH + CM group (22.8 ± 8.9, range: 3–40 years) was significantly higher (*p* < 0.001) than EM group (10.8 ± 6.9, range: 0.83–20 years). The medication-overuse duration of MOH + CM subjects was 7.3 ± 6.4 with range of 0.25–17 years. The visual analog scale of MOH + CM and EM group resulted no significant difference. The PHQ-9 depression scale of MOH + CM patients (8.7 ± 6.7, range: 0–23) was significantly higher than EM (2.4 ± 4.8 range: 0–11) and HC (2.5 ± 2.2, range: 0–6), with *p* value of 0.018 and 0.002 separately. There was no difference of GAD-7 anxiety scale of participants in three groups. Overused medications were combination analgesics for all MOH + CM participants (chiefly aminopyrine-phenacetin-phenobarbital-caffeine and caffeine-aminopyrine).

**Table 1:** Demographic and clinical data for all participants

Clinical details	MOH + CM^a^ patients	EM^b^ patients	Healthy controls	*p* value
Age, years	46.6 ± 10.3	38.0 ± 14.4	40.8 ± 9.7	0.078
Sex	3M^c^, 14F^d^	4 M, 14 F	11 M, 19 F	0.298
Headache history, years	22.8 ± 8.9	10.8 ± 6.9	N/A^e^	< 0.001
Medication-overuse duration, years	7.3 ± 6.4	N/A	N/A	N/A
Headache location				
Right; Left; Bilateral	2 (11.8 %); 2 (11.8 %); 13 (76.5 %)	2 (11.1 %); 7 (38.9 %); 9 (50 %)	N/A; N/A; N/A	N/A; N/A; N/A
VAS^f^	8.4 ± 1.4	8.0 ± 1.4	N/A	0.465
PHQ9	8.7 ± 6.7	2.4 ± 4.8	2.5 ± 2.2	< 0.05^h^
GAD-7^i^	2.9 ± 3.9	1.6 ± 3.6	2.6 ± 2.1	0.729

^a^ MOH + CM, medication-overuse headache + chronic migraine; ^b^ EM, episodic migraine; ^c^ M, male; ^d^ F, female; ^e^ NA, not applicable; ^f^ VAS, visual analog scale; ^g^ PHQ-9, Patient Health Questionnaire 9 Depression Scale; ^h^ Post-hoc analysis using LSD resulted significant difference between MOH + CM and EM with *p* value of 0.018, MOH + CM with HC with *p* value of 0.002. Meanwhile there was no significant difference between EM and HC with *p* value of 0.978; ^i^ GAD-7, Generalized Anxiety Disorder Assessment 7-item Scale. Data are mean ± standard deviation

### GICA spatial maps

**Fig. 2:**
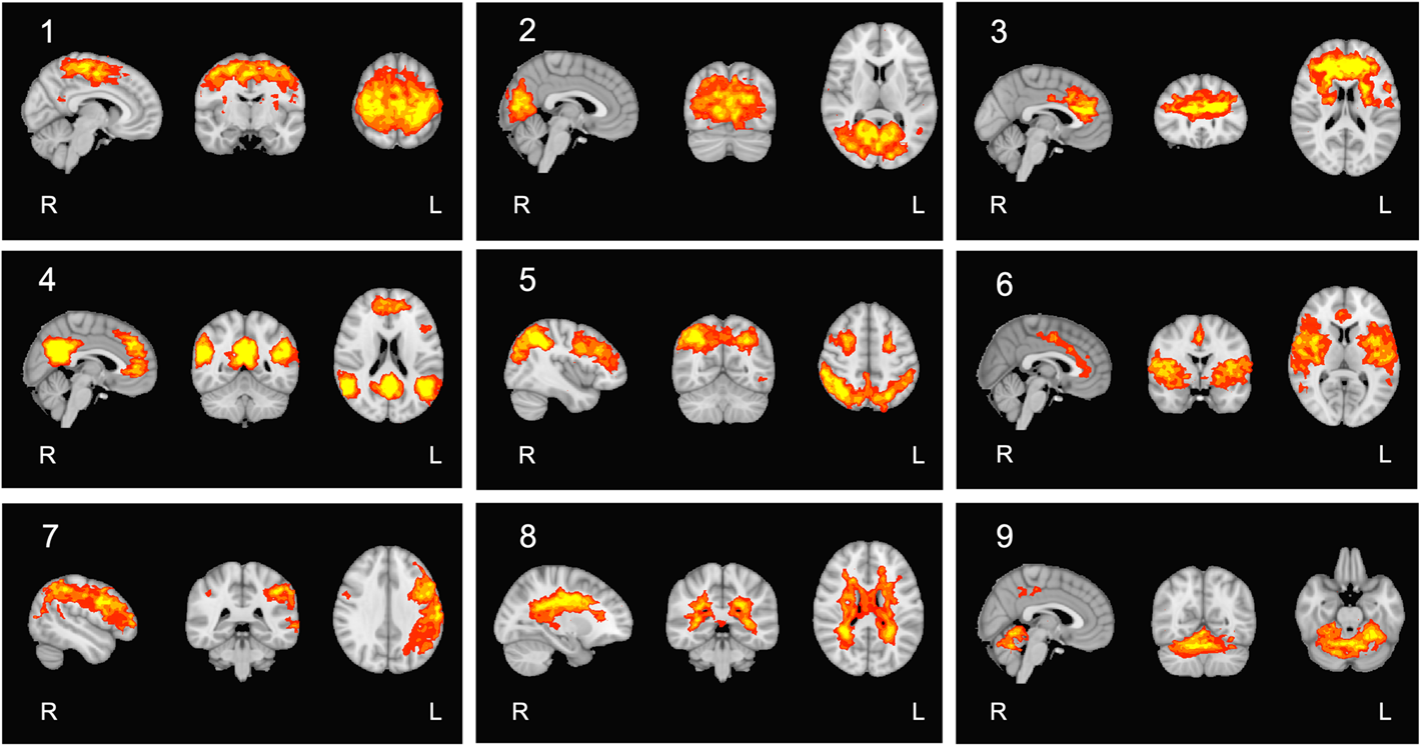
Group ICA–estimated resting-state networks based on 50-component analysis. Sagittal, axial, and coronal views of the ICA spatial maps estimated by correlation to the known reference network using FSL utility and confirmed by visual inspections, considering 9 nonartifactual components

RS-fMRI data were separated from the entire HC group into 50 independent components. We obtained 9 nonartifactual components based on correlation analysis results and visual inspection of each component’s spatial map (Fig. 2). Then we selected the SN on the basis of the r-value and spatial similarity to the reported spatial maps previously [32].

### Altered resting-state interaction of the habenula and the SN in patients with MOH + CM

**Fig. 3:**
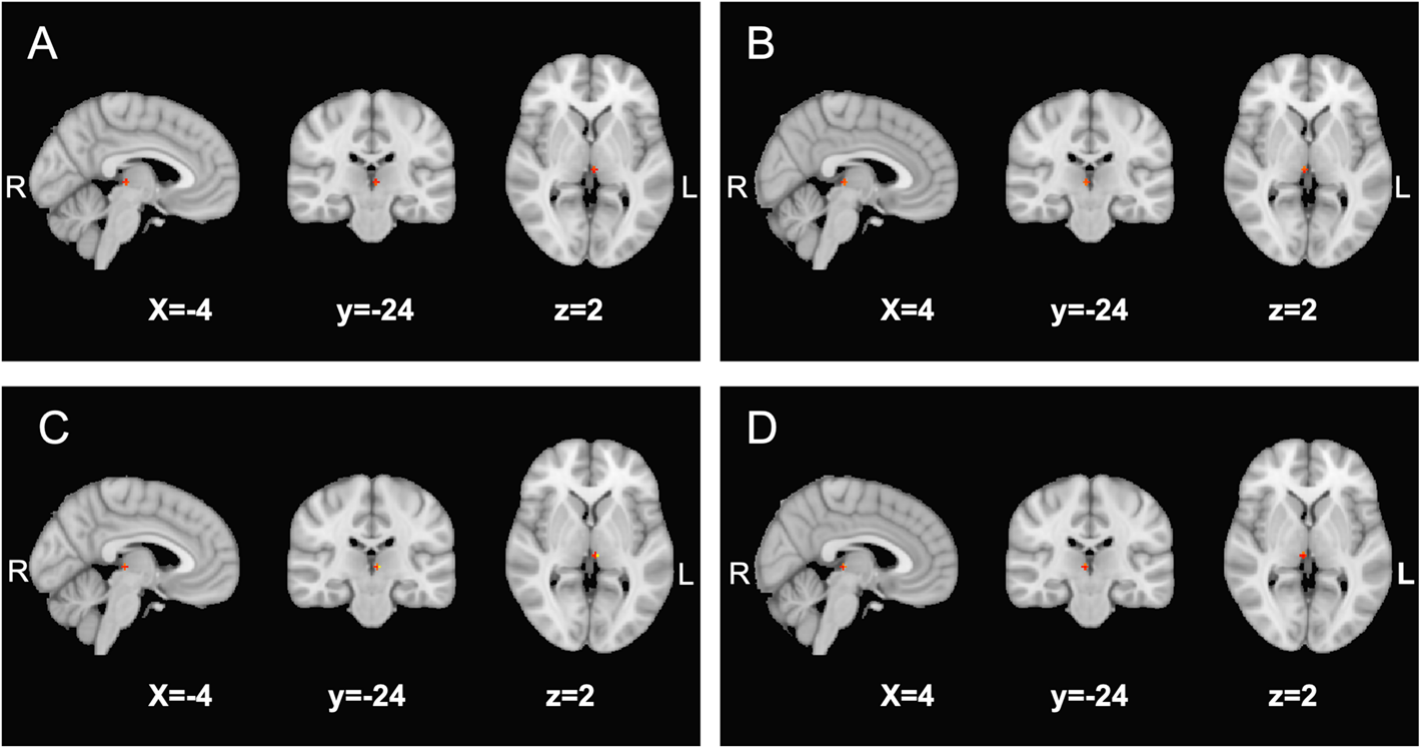
Altered habenula-SN functional connectivity maps. Altered resting-state functional connectivity of habenula and the SN region in MOH + CM vs. that in HC and EM patients. (**A**) the increased resting-state functional connectivity of the left habenula and the SN in patients with MOH + CM compared with HC; (**B**) the increased resting-state functional connectivity of the right habenula and the SN in patients with MOH + CM compared with HC; (**C**) the increased resting-state functional connectivity of the left habenula and the SN in patients with MOH + CM compared with EM; and (**D**) the increased resting-state functional connectivity of the right habenula and the SN in patients with MOH + CM compared with EM. SN, salience network; MOH + CM, medication-overuse headache + chronic migraine; HC, healthy controls; EM, episodic migraine

We identified an increased functional connectivity of habenula-SN in both the right and left habenula masks of the MOH + CM subjects compared with EM and HC. While there was no difference detected between EM subjects and HC in the same brain regions. Further details are shown in Table 2; Fig. 3. No difference was detected between subgroups with or without depression based on PHQ-9 scale in MOH + CM (Table 2).

**Table 2:** Altered resting-state functional connectivity of the habenula and the SN^a^ amongst MOH + CM^b^, EM^c^ and HC^d^, and between MOH + CM subgroups with or without depression

Brain region	Number of voxels	Cluster size, mm^3^	MNI coordinates, mm			F score (max)	*p* value
			x	y	z		
MOH + CM VS HC							
R^e^ habenula	7	56	4	-25	2	3.38	< 0.001
L^f^ habenula	7	56	-3	-25	2	3.09	0.002
MOH + CM VS EM							
R habenula	7	56	4	-23	2	2.73	0.006
L habenula	7	56	-3	-23	2	3.03	0.002
EM VS HC							
R habenula	0	0	3	-25	2	1.76	0.079
L habenula	0	0	4	-24	2	1.51	0.132
MOH + CM ^depression^ VS MOH + CM ^without depression^							
R habenula	0	0	5	-24	2	0.85*	0.475
L habenula	0	0	4	-24	2	1.99*	0.077

^a^ SN, salience network; ^b^ MOH + CM, medication-overuse headache + chronic migraine; ^c^ EM, episodic migraine; ^d^ HC, healthy controls; ^e^ R, right; ^f^ L, left

*T score from two-sample t test

### Correlation analysis between habenula-SN functional connectivity and clinical variables

**Fig. 4:**
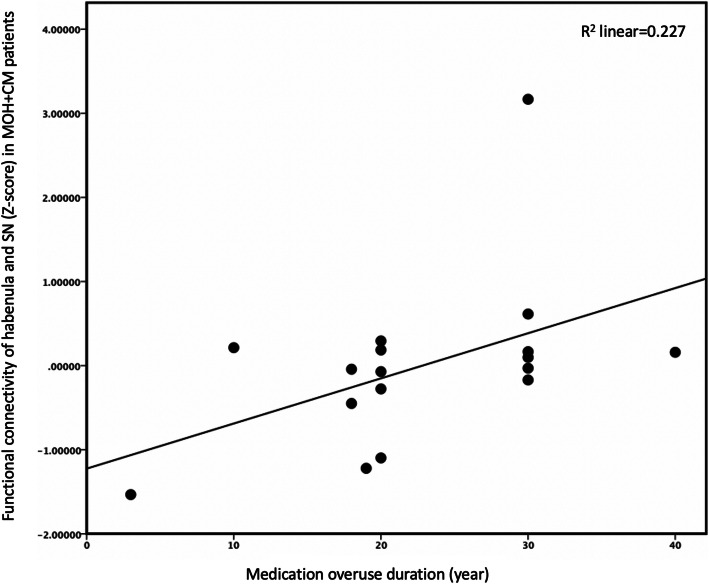
Correlation analysis between functional connectivity of habenula and SN and medication overuse duration. Correlation analysis results of the functional connectivity between habenula and SN and medication overuse duration. The relationship between these resting-state abnormalities and the other clinical variables were also checked. No results exceeded the threshold. SN, salience network

Table 3; Fig. 4 presented that there was significant correlation between SN-habenula functional connectivity and medication overuse over time (*p* = 0.043, *r* = 0.512, 95 %CI = 0.116–0.790). There were no significant correlations between SN-habenula connectivity and other clinical variables (*p* > 0.05).

**Table 3:** Correlation analysis between functional connectivity of habenula-SN and clinical variables of MOH + CM^a^ subjects

Clinical variables	r	*p* value	95 % CI of r
Headache history	0.103	0.703	-0.204–0.557
Medication overuse duration	0.512	0.043	0.116–0.790
Attack frequency	0.299	0.260	-0.451–0.702
VAS^b^	0.205	0.446	-0.300–0.714
PHQ-9^c^	0.482	0.158	-0.333–0.882
GAD-7^d^	0.206	0.568	-0.488–0.659

^a^ MOH + CM medication-overuse headache + chronic migraine; ^b^ VAS visual analog scale; ^c^ PHQ-9 Patient Health Questionnaire 9 Depression Scale; ^d^ GAD-7 Generalized Anxiety Disorder Assessment 7-item Scale

## Discussion

To the best of our knowledge, it is the first time that functional connectivity between the habenula and the SN in patients with MOH + CM and EM were investigated. Based on the independent component analysis (ICA) method, bilateral habenula showed increased resting-state functional connectivity with the SN in MOH + CM patients compared with EM and HC. Our results suggested this aberrant enhanced functional connectivity may play an important role in MOH and provided understanding for the pathophysiology of this condition.

So far, the pathophysiology of MOH has been believed to be associated with the central sensitization, deficit endogenous pain modulation, genetic predisposition, and substance addiction [6, 33, 34]. As for MOH, the chronic pain itself is a complicated and multidimensional sensory experience that includes three domains: sensory, cognitive, and affective, which overlap largely with the function of the habenula and the SN [35].

In the current study, the increased functional connectivity of habenula-SN was shown in patients with MOH and CM but not in EM or HC, or between subgroup of MOH + CM patients with or without depression, which indicated that this alteration may correlate with either repetitive headache attacks or overuse of medication. Previous studies have demonstrated increased functional connectivity and decreased gray matter volume within SN in MOH patients compared with healthy individuals [9, 36], indicating the involvement of SN in MOH. In MOH patients who evolved from migraine, the dACC was demonstrated to be overactivated during switch between noxious and innocuous stimuli, yielding dysregulated salience processing of sensory in chronic migraine, and this overactivation to unspecific salient stimuli may play a role in migraine chronification [37]. On the other hand, habenula activation has been observed in noxious stimulation together with increased functional connectivity with periaqueductal grey [38]. The increased synchronous neuronal activity of habenula-SN is likely to correlate with the MOH processing and might contribute to the MOH development or chronification through aberrant overactivation to unspecific sensory stimuli.

MOH is hypothesized to be lying within a spectrum of addiction disorder involving the mesocorticolimbic dopamine system [39]. As a core component of the brain anti-reward system, the lateral habenula receives input from limbic-forebrain and basal ganglia and sends output to midbrain nucleus including ventral tegmental area (VTA) and substantia nigra compacta (SNc) [40]. The habenula is activated by exposure to negative reinforces such as pain or stress and provides inhibitory tone to decrease the activity of VTA and SNc. This will turn off the reward system and results in reduced dopamine release [41, 42]. As for MOH which evolves from a pre-existing headache, presumably the repetitive headache attacks may gradually reduce dopamine release through habenula during the process. The habenula-dopamine pathway was proved to motivate anticipation through the integrity of phasic salience-related signals and tonic reward-related signals [43]. And the insula of SN was proved to charge in the conscious desire for drugs for it role in incentive motivation [44]. Taken together, this habenula-SN dysfunction is likely to intervene with cognitive control to take the medications and fail to integrate motivational information in the long-term and may play a role in the development or the aggravation of MOH. Meanwhile, the increased functional connectivity of habenula-SN showed significant correlation with medication overuse duration, which indicated that the time length of medication overuse was related to the enhanced synchronous neuronal activity of habenula and SN. Furthermore, the habenula might be an efficient indicator or treatment target for MOH for its integrative role that covering multiple aspects of MOH.

## Limitations

Our study has several limitations. First, the sample size was limited. Second, the participants were not matched according to the depression or anxiety scale since the highly presence of depression in MOH population in the real world. Although PHQ-9 and GAD-7 scale were used as covariates to minimize these effects, and further subgroup comparisons within MOH patients were performed based on two scales, we cannot definitely exclude the possibility that part of the functional connectivity changes may be due to depression or anxiety. Third, we were not able to draw a causative relationship of the increased functional connectivity of habenula-SN and the MOH development. Fourth, we were not able to compare the habenula-SN functional connectivity amongst patients overusing different medications in MOH, since all our participants had overused combination analgesics. Previous studies based in China showed patients who overused combination analgesics and more than two kinds of analgesics accounted for almost 90 % of MOH [2, 45]. While triptan, ergotamine and opioid were rarely used either for its relatively high cost as a prescription only available in a few hospitals, or for its side effect and no longer available in the market, or strictly constricted [2]. Meanwhile combination analgesics were available as over the counter at a relatively low price. Further studies are needed to clarify the effects of headache medications per se on the entity-specific alterations in the brain.

## Conclusions

The current study demonstrated an increased functional connectivity of habenula-SN in patients with MOH + CM compared with EM and HC by ICA-based analyses of RS-fMRI data. Our findings supported MOH to be lying within a spectrum of dependence and addiction disorder. The enhanced functional connectivity of the habenula with SN may correlate to the development or chronification of MOH. Furthermore, the habenula may be an indicator or treatment target for MOH for its integrative role involved in multiple aspects of MOH.

## Data Availability

All data generated and analyzed during the current study will be available from the corresponding author on reasonable request.

## Abbreviations

MOH: Medication-overuse headache
CM: Chronic migraine
RS-fMRI: Resting-state functional magnetic resonance imaging
SN: Salience network
EM: Episodic migraine
HC: Healthy controls
PHQ-9: Patient Health Questionnaire 9 Depression Scale
GAD-7: Generalized Anxiety Disorder Assessment 7-item Scale
EPI: Echo-planar imaging
FSL: FMRIB’s Software Library
BET: Brain Extraction Tool
FWHM: Full width at half maximum
TC-GICA: Temporal-concatenation group independent component analysis
MELODIC: Multivariate Exploratory Linear Optimized Decomposition into Independent Components
ICA: Independent component analysis
FDR: False discovery rate
VTA: Ventral tegmental area
SNc: Substantia nigra compacta
dACC: Dorsal anterior cingulate cortex
